# Integrating CRISPR-Cas and Next Generation Sequencing in Plant Virology

**DOI:** 10.3389/fgene.2021.735489

**Published:** 2021-10-25

**Authors:** Muntazir Mushtaq, Aejaz Ahmad Dar, Umer Basu, Basharat Ahmad Bhat, Rakeeb Ahmad Mir, Sanskriti Vats, M. S. Dar, Anshika Tyagi, Sajad Ali, Monika Bansal, Gyanendra Kumar Rai, Shabir Hussain Wani

**Affiliations:** ^1^ Division of Germplasm Evaluation, ICAR-National Bureau of Plant Genetic Resources, New Delhi, India; ^2^ School of Biotechnology, Sher-e-Kashmir University of Agricultural Sciences and Technology of Jammu, Jammu, India; ^3^ Division of Plant Pathology, Sher-e-Kashmir University of Agricultural Sciences and Technology of Jammu, Jammu, India; ^4^ Department of Bioresources, University of Kashmir, Srinagar, India; ^5^ Department of Biotechnology, School of Biosciences and Biotechnology, BGSB University, Rajouri, India; ^ **6** ^ Department of Agricultural Biotechnology, National Agri-Food Biotechnology Institute (NABI), Mohali, India; ^7^ Division of Plant Pathology, Sher-e-Kashmir University of Agricultural Sciences and Technology of Kashmir, Srinagar, India; ^8^ Department of Biotechnology, Yeungnam University, Gyeongsan, South Korea; ^9^ School of Agricultural Biotechnology, Punjab Agricultural University, Ludhiana, India; ^10^ Mountain Research Centre for Field Crops, Sher-e-Kashmir University of Agricultural Sciences and Technology of Kashmir, Srinagar, India

**Keywords:** virus, viroid, NGS, genome editing, CRISPR

## Abstract

Plant pathology has been revolutionized by the emergence and intervention of next-generation sequencing technologies (NGS) which provide a fast, cost-effective, and reliable diagnostic for any class of pathogens. NGS has made tremendous advancements in the area of research and diagnostics of plant infecting viromes and has bridged plant virology with other advanced research fields like genome editing technologies. NGS in a broader perspective holds the potential for plant health improvement by diagnosing and mitigating the new or unusual symptoms caused by novel/unidentified viruses. CRISPR-based genome editing technologies can enable rapid engineering of efficient viral/viroid resistance by directly targeting specific nucleotide sites of plant viruses and viroids. Critical genes such as eIf (iso) 4E or eIF4E have been targeted via the CRISPR platform to produce plants resistant to single-stranded RNA (ssRNA) viruses. CRISPR/Cas-based multi-target DNA or RNA tests can be used for rapid and accurate diagnostic assays for plant viruses and viroids. Integrating NGS with CRISPR-based genome editing technologies may lead to a paradigm shift in combating deadly disease-causing plant viruses/viroids at the genomic level. Furthermore, the newly discovered CRISPR/Cas13 system has unprecedented potential in plant viroid diagnostics and interference. In this review, we have highlighted the application and importance of sequencing technologies on covering the viral genomes for precise modulations. This review also provides a snapshot vision of emerging developments in NGS technologies for the characterization of plant viruses and their potential utilities, advantages, and limitations in plant viral diagnostics. Furthermore, some of the notable advances like novel virus-inducible CRISPR/Cas9 system that confers virus resistance with no off-target effects have been discussed.

## Introduction

Plant viral diseases present the most central challenge to twenty-first century agriculture systems on a global scale. Viruses are recognized to cause destructive plant diseases which lead to considerable losses in terms of yield as well as quality in the majority of crop plants worldwide (Mushtaq et al., 2020; Rubio et al., 2020). The projected cumulative crop damage caused due to pathogens is up to 15%, out of which viruses are instrumental and contribute 47% loss in the total yield (Boualem et al., 2016; Mushtaq et al., 2020). The global cost of controlling infections in cultivated crops due to viruses is anticipated to be higher than US $30 billion per year (Nicaise, 2014; Sastry and Zitter, 2014; Cao et al., 2020). Viral infection in crops has intensified at an unprecedented speed because of climate change, global warming, the increasing food demands of the human population, and the movement of insect vectors are causing dramatic changes in farming practices and cropping systems that encourage the spread of catastrophic viral disease outbreaks (Trębicki et al., 2016; Mushtaq et al., 2020). In food-insecure countries, such epidemics are especially visible in subsistence agriculture. (Jones and Naidu, 2019). Agricultural explosive growth and rapid international trade expansion of plants and plant produce has contributed to the movement of viral diseases and disseminated them to wide geographical regions to mediate unpredictable implications on the ecosystem and food production (Mushtaq et al., 2020).

Owing to the unpredictable epidemiological nature of various virus pathosystems, there is not any versatile method to mitigate the harmful effects of viral diseases on different agro-ecosystems. Advances in technical expertise focusing on virus pathosystems, accelerated scientific progress, ground-breaking connectivity plans, and global logical networks create an incentive to develop epidemiological virus suppression intelligence for agricultural development and overall food security. A paradigm shift towards the production of interconnected, smart, and sustainable solutions is required to advance the management of virus diseases in various cropping systems.

Plant viruses are obligate intracellular parasites, which have limited coding capacity and rely on the host plant for completing their life cycle. Unlike other plant diseases, there are hardly any successful remedies to cure harmful plant viruses without devising a specialized strategy. Consequently, plant molecular breeding is being considered as an indispensable tool to generate immunity, resistance, or tolerance to plant viruses in order to improve agricultural production.

An effective strategy to check viral attacks entails useful detection methods and thereafter deliberating the insights into the targeted viral genomes. The initial screening tools include PCR-based techniques such as RT-PCR and other variants. These diagnostic tools need prior knowledge of viral genomes and as a result, it ends up exhibiting poor detection of viruses with little information regarding their genomes (Shahid et al., 2021). So far, at least 1,500 plant virus species (26 families) have been known and characterized based on the genomic sequences of the viruses (Cao et al., 2020). Against these drawbacks, next-generation sequencing (NGS) may serve as an unbiased technology for the diagnosis of plant viral diseases, since no prior information about the pathogen is required. With this technology, plant virology is closely bridged with molecular biology through in-depth genomic information, leading to precise targeting of viral pathogens with significant improvement over existing technologies. Present-day NGS tools are capable of sequencing any type of nucleic acid, concomitantly. NGS technologies have emerged as the tool of choice to detect novel viral diseases from very few viral titers (Villamor et al., 2019). Therefore, with this technology, our understanding regarding phytoviromes has expanded horizons to facilitate future targeted approaches which will readily achieve their desired results.

Genome editing technologies have evolved to induce specific and targeted modifications into the plant genome to obtain desired results, such as the development of next-generation plant breeding through precision breeding systems.

The evolution of higher organisms is highly augmented by gene-editing technologies, such as Meganucleases, Zinc Finger Nuclease (ZFN), Transcription Activator like Effector Nuclease (TALEN), and CRISPR-Cas9 systems (Wiedenheft et al., 2011; Jinek et al., 2013; Zhang et al., 2013; Shahid et al., 2021). Amongst these technologies, the most recent CRISPR-Cas system imparts several advantages such as precise and flexible genome editing at the preferred genomic site to induce desirable mutations (Bortesi and Fischer, 2015). The CRISPR/Cas system has evolved as the leading and pioneering technology to edit genomes across all the kingdoms, although plant genome editing experiments were successfully carried out for the first time in 2013 (Li et al., 2013; Nekrasov et al., 2013; Shan et al., 2013). Since then, CRISPR/Cas mediated genome editing in plants has increased at a fast rate in contrast to the rest of new plant breeding technologies (NPBTs). CRISPR tool is based on RNA-programmed DNA cleavage systems, which were revealed in bacteria and archaea (Hsu et al., 2014). The last decade witnessed several reports regarding the diverse working principles of CRISPR-Cas based genome editing, especially the CRISPR/Cas9 system (Chen et al., 2019; Hanna and Doench, 2020).

**FIGURE 1:**
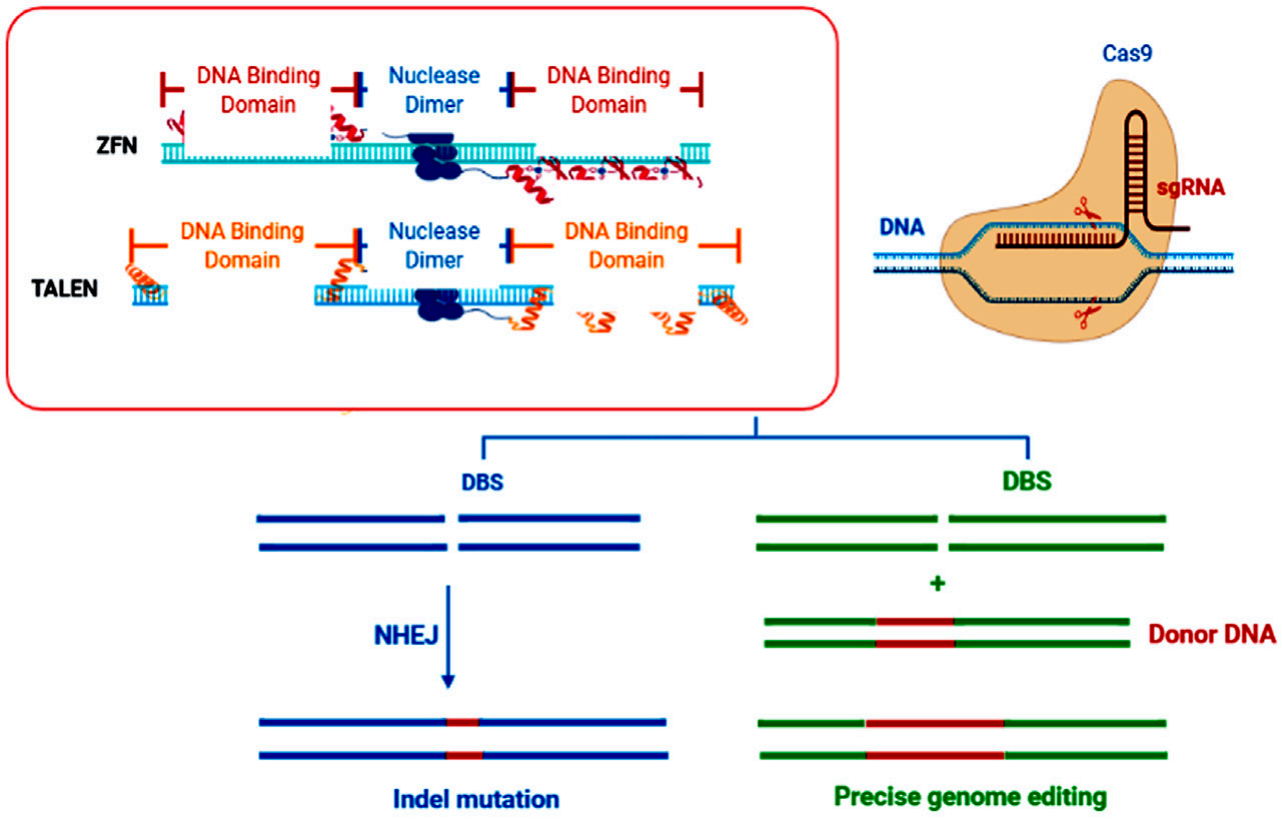
Schematic diagram of the NHEJ and HDR DNA repair pathways when DNA double-strand breaks (DSBs) are produced by sequence-specific nucleases (SSNs). CRISPR-based genome editing takes advantage of Cas9’s ability to induce targeted DNA double-strand breaks (DSBs) usually a few nucleotides upstream of the PAM sequence. The chromosomal DSBs are repaired by the cell via two repair pathways, either non-homologous end joining (NHEJ) or homology-directed repair (HDR). At its core, NHEJ-break ends can be ligated without a homologous template, while HDR-breaks require a template to guide repair.

In brief, CRISPR-Cas9 was found for the first time in *Streptococcus pyogenes* and reported as a type II immune system of prokaryotes against invading bacteriophages (Jinek et al., 2012). The later system relies on double-strand breaks (DSBs) induced at specific sites in the invading viral DNA. Consequently, DSBs trigger a DNA-repair mechanism in host cells through homology-directed repair (HDR) or non-homologous end-joining (NHEJ) (Figure 1) and induces insertions or deletions (indels) in the target viral DNA to make it non-functional against the host bacteria (Zaidi et al., 2020).

In an engineered CRISPR system, at the CRISPR locus a small CRISPR RNA (crRNA) is transcribed, which hybridizes with target genomic sequences through a complementary sequence present on the sequence flanking protospacer-associated motif site (PAM). The canonical 5′-NGG-3′ PAM is important in order to be recognized by the Cas9 protein for recognition and action in the target viral genome to induce immunity in *Streptococcus pyogenes* (Wright et al., 2016). Subsequently, a considerable portion of crRNA binds to trans-activating RNA (tracrRNA), and both bind to Cas9-gRNA complex to form a complete genome editing machinery. This complex now binds to complementary target sites in the target genome through gRNA and then Cas9 nuclease induces DSBs almost three nucleotides upstream of the PAM site (O’Connell et al., 2014). Accordingly, this system is therefore capable of generating precise, site-specific alterations in DNA via the synthetic single guide (sg) RNAs designed to direct Cas9-mediated cleavage at targeted sites (Hanna and Doench, 2020). The only criterion for CRIPSR to be used against the targeting of genes lies in the presence of a protospacer-adjacent pattern (PAM) sequence near the target site (Gleditzsch et al., 2019). Using CRISPR gene-editing for different targets requires only different spacer sequences; thus, it is quick, easy, effective, economical, and scalable (Zhang et al., 2020).

**FIGURE 2:**
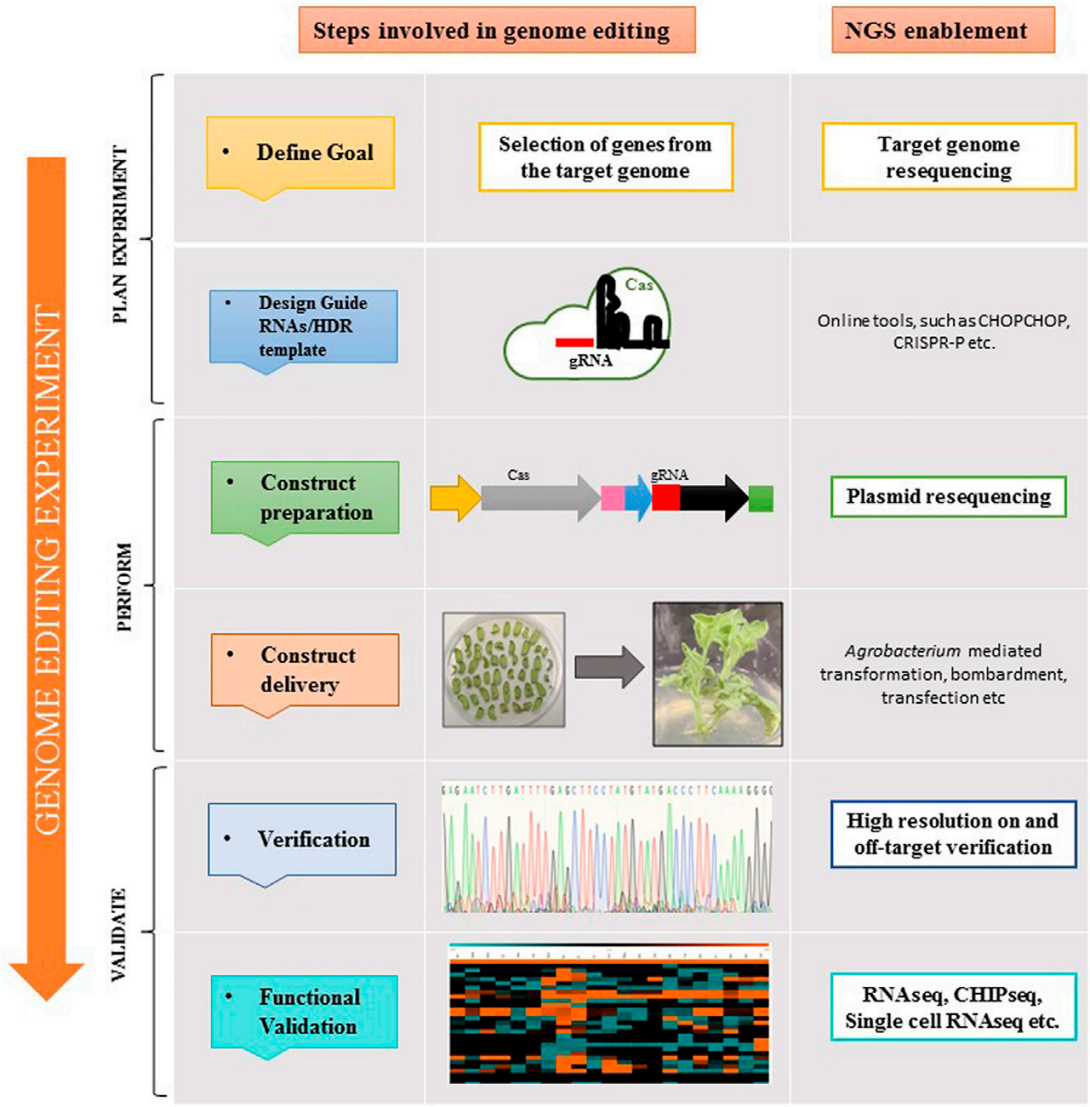
Application of NGS at different steps of the plant genome editing workflow. The first step in designing a CRISPR experiment is selecting the suitable CRISPR-associated (Cas) enzyme. The protospacer-adjacent motif (PAM) sequence determines which Cas enzyme to use because it ascertains potential target sites for genome editing. In order to direct the Cas enzyme to the target site in the genomic DNA, guide RNA (gRNA) is optimally designed. For Cas9, the gRNA can be made either as a single guide RNA (sgRNA) or a 2-part guide RNA (containing crRNA and tracrRNA). To deliver Cas enzyme and guide RNA to cells, agrobacterium-mediated transformation, bombardment, transfection, etc. can be used. For determining the success of on-target editing and for examining off-target effects, NGS is highly recommended.

NGS is indispensable for genome editing experiments as well, especially clustered regularly interspaced short palindromic repeats (CRISPR)/CRISPR-associated (Cas) based gene editing. From validating (CRISPR) knockouts to examining off-target effects or other edits with targeted sequencing, NGS is employed at different steps of the genome editing workflow (Figure 2). Follow-up research can then be carried out using applications, for example, methylation and gene expression analysis with RNAseq to assess the functional impact of a certain gene edit (Bhat and Rao, 2020).

**FIGURE 3:**
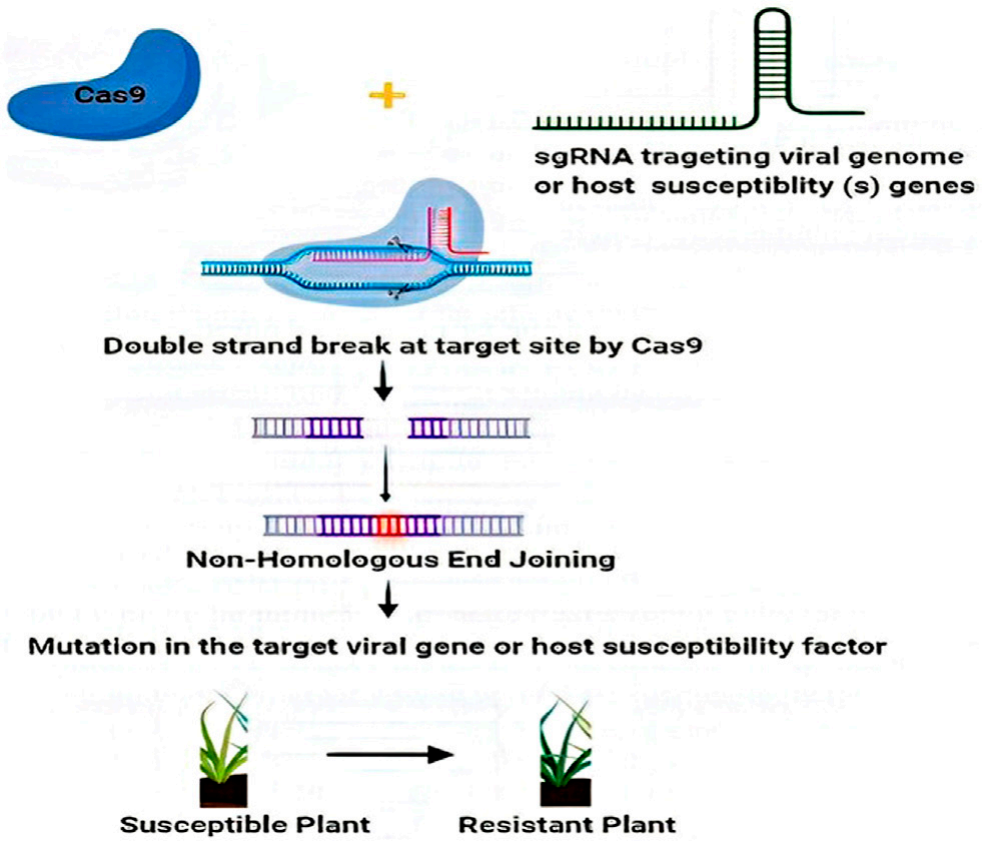
Utilization of CRISPR/Cas systems against diverse plant viruses by targeting host susceptibility genes or viral genome. The viral genome DNA or RNA can be targeted, destroyed, or interfered with by CRISPR/Cas9 in the nucleus or in the cytoplasm, thus inhibiting plant viruses by CRISPR/Cas9 system-mediated adaptive immunity. Additionally, the mutation in host susceptibility factors edited by the CRISPR/Cas9 system further contributes to viral interference.

Contemporary advances in CRISPR/Cas based genome editing render it a desirable tool for developing or inducing plant defense. Two major pathways are employed by CRISPR/Cas systems to enhance the virus resistance in crop plants. The first way is the CRISPR/Cas-mediated targeted mutagenesis of specific genes in host plant responsible for contributing to the viral cycle, and second, CRISPR/Cas systems can be configured to work efficiently in plants for targeting viral genomes (Mushtaq et al., 2019; Kalinina et al., 2020; Zhao et al., 2020; Shahid et al., 2021). For instance, CRISPR/Cas9 systems could be used to directly target viruses with DNA as well as RNA genomes, while other CRISPR/Cas systems such as, Cas13a (Abudayyeh et al., 2017) and Cas9 from *Franciscella novicida* (FnCas9) (Price et al., 2015) could specifically target viruses which have RNA genomes (Green and Hu, 2017; Wolter and Puchta, 2018). In this review, we discuss the applications of CRISPR/Cas systems against diverse plant viruses by targeting the susceptible genes of the host or viral genomes (Figure 3), and additional advancements in this particular field. Further, we register certain possible recessive resistance genes which can be exploited in antiviral breeding programs and highlight the relevance of antiviral breeding based on recessive-resistance genes to produce virus-free plants. Finally, we address the problems and landscape for applications of CRISPR/Cas technology for the avoidance and management of plant viruses/viroids in the field. Overall, this review provides a snapshot vision of the role of NGS and spectacle applications of CRISPR-Cas editing technologies in plant virology.

## Next-Generation Sequencing Technologies as Go-To Tool for Plant Virology

Several technological breakthroughs have been employed to overcome the precise detection of plant viromes. One among these modern technologies is Next-generation sequencing (NGS), a novel tool for viral detection in diseased plants. In 2009, NGS technologies were initiated in plant virology for genome sequencing, discovery and identification, and epidemiology and ecology of viruses and viroids (Adams et al., 2009). The NGS pipeline involves the isolation of total RNA, DNA, or small RNA (sRNA) from the infected plant, the synthesis of cDNA and sequencing, then analyzing the sequencing data, contig development, and blasting the contigs sequence against a plant virus database assists in the recognition and characterization of target viruses. The detection and characterization of unknown and unidentified viruses/viroids from infected plants are probably the major promising application of such technologies (Table 1) (Barba et al., 2014). The RT-PCR helps to validate the NGS results using the complete RNA of the infected plant (Bhat and Rao, 2020). NGS provides a breakthrough to study viral diversity at taxonomic hierarchy levels. Its wide computational analyses by many programs and algorithms have created exciting opportunities for virus diagnostics and discovery. Unfolding evolutionary dynamics of viruses enhances the understanding of quasispecies diversity and the involvement of mutations in drug resistance and host switching, enabling the genotypic and phenotypic characterization of viruses (Kasibhatla et al., 2016).

**TABLE 1:** List of viruses/viroids detected by NGS.

S.No.	Viruses/viroids identified	Host plant	Sequencing platform	Sequencing target	Reference
1.	Emaravirus	*Acer pseudoplatanus*	Illumina HiSeq2500	Total RNA	Rumbou et al. (2021)
2.	PSTVd	*Solanum lycopersicum*	Illumina MiSeq sequencer	vd-sRNA	Adkar-Purushothama et al. (2020a)
3.	GaP1V1 and Grapevine cogu-like virus	*Vitis* spp.	Illumina Novaseq technology	Total RNA	Bertazzon et al. (2020)
4.	GFLV, GaCLV-1, GaCLV-2, GaCLV-3, GLRaV-1, GLRaV-3, GaIV-1, Entoleucaphenui-like virus 1, GaJV-1, GaJV-2 and PaPLV 1	*Vitis vinifera*	Illumina HiSeq	Total RNA and ribo-depleted RNA	Chiapello et al. (2020)
5.	Tomato yellow leaf curl Kanchanaburi virus, AYVMV	*Solanum lycopersicum* cv.MT1	Illumina Hiseq 2000	DNA	Mahmoudieh et al. (2020)
6.	23 viruses and viroids (including grapevine leafroll associated virus 4)	*Vitis vinifera*	Illumina NextSeq500 platform	mRNA and sRNA	Sidharthan et al. (2020)
7.	AV-2, BBWV-2, GaILV, CMV, TSWV and LNRSV	*Valeriana fauriei* Briq	-	Total RNA	Uehara-Ichiki et al. (2020)
8.	CaYRSV, CaCRSVs, CaBaV, and (CaMaV)	*Camellia japonica*	Illumina HiSeq X-ten platform	Total RNA	Zhang et al. (2020)
9.	PVM	*Solanum lycopersicum*	Illumina MiSeq platform along with High-throughput sequencing (HTS) and Sanger sequencing	Total RNA	Glasa et al. (2019)
10.	GLRaV-1, GRSPaV, GRGV and GYSVd1, GVA and Grapevine Syrah virus 1, HSVd	*Vitis berlandieri* × *V. riparia* ‘Kober 125AA and	-	sRNA	Massart et al. (2019)
	PVX and PVB	*Solanum tuberosum*			
	ASGV	*Malus* × *domestica* ‘Golden Delicious			
11.	CVT	*Prunus avium* L.	Illumina HiSeq 2500	Total RNA	Milusheva et al. (2019)
12.	PLMVd, APV1, PBNSPaV, CNRMV, CGRMV, CNRMV, NSPaV, ACLSV, and PeVD	*Prunus persica*cv. *Nectarina*	Illumina HiSeq. 2500 SE50	sRNA	Xu et al. (2019)
13.	DBRTV3-[2RT] and DBRTV3-[3RT]), YMV-NG	*Dioscorea* spp.	Illumina HiSeq4000	Total RNA	Bomer et al. (2018)
14.	CMV, ANSV and PYVV	*Solanum quitoense*	llumina HiSeq-2500	Total RNA and ribo-depleted RNA	Gallo et al. (2018)
15.	SMV	*Passifloraedulis*	llumina HiSeq-2500	Total RNA and ribo-depleted RNA	Jaramillo et al. (2018)
16.	HaEV	*Helianthus annuus*	Illumina HiSeq 2500	sRNA	Liu et al. (2018)
17.	GRSPaV, GVB, GFkV, GLRaV-3 and HSVd	*Vitis vinifera*	Illumina HiSeq2000	sRNAs	Barrero et al. (2017)
	PNRSV RNA1, PNRSV RNA2 and PNRSV RNA3	*Prunus persica*			
	(RBDV) RNA1, RBDV RNA2 and (RYNV)	*Rubusidaeus*			
	(RNA dependent RNA polymerase) [PCV (RdRp)] and Arisoteliachilensis virus (Reverse transcriptase) [ACV (RT)]	*Brassica sp.*			
	SPSMV-1	*Ipomoea batatas*			
	(SMoV) RNA1 and SMoV RNA2	*Fragaria ananassa*			
	SrMV	*Miscanthus sinensis*			
	CTV, CVd-VI and HSVd	*Citrus medica*			
	Citrus endogenous pararetrovirus	*Citrus sp.*			
	MCDV	*Pennisetum advena*			
	CTV	*Citrus latifolia*			
	AcVB	*Actinidia*			
	PVY and TVCV	*N. tabacum*			
18.	14 viral/viroid species detected by both sRNA and rRNA method. But a novel viral species from the CcyV1 genus was detected only by rRNA depleted totRNA approach	*Solanum tuberosum, Solanum lycopersicum, Brassica oleracea, N. tabacum, N. benthamiana, P. sativum* and *Prunu s*sp.	Illumina HiSeq 2500 (United States)	sRNA versus rRNA	Pecman et al. (2017)
19.	342 viruses/viroid sequences	*P. domestica*, *Prunusavium*, *P. persica*, *Pyrus communis and Malus domestica*	Illumina HiSeq 2500	dsRNA	Rott et al. (2017)
20.	PVY,PeMV, BrYV, TMV, TVBM, CVMV, BBWV2, CMV	*N. tabacum*	Illumina HiSeq-2000	Total RNA	Akinyemi et al. (2016)
21.	PlAMV	*Viola grypoceras* and *Nandina domestica* Thunb	-	Total RNA	Komatsu et al. (2016)
22.	GLRaV-3 and HSVd	*Vitis vinifera*	Illumina HiSeq	sRNA and ribo-depleted RNA	Visser et al. (2016)
	CTV and CDVd	*Citrus paradisi*			
23.	PPSMV	*Cajanus cajan*	Illumina technology	Total RNA	Elbeaino et al. (2014)
24.	BYMV and ClYVV	*Lupinus angustifolius*	Illumina HiSeq2000	Total RNA	Kehoe et al. (2014)
25.	LChV1	Prunus *avium*	Roche 454 Pyrosequencing	dsRNA	Candresse et al. (2013)
26.	4 variants of PVY	Capsicum annuum L.	Roche 454	Total RNA	Fabre et al. (2012)
27.	GRSPaV,GSyV-1, HSVd and GYSVd1, GRVFV	Vitis vinifera	Illumina Genome Analyzer II	sRNAs	Giampetruzzi et al. (2012)
28.	Raspberry latent virus	Rubusidaeus	Illumina	dsRNA	Quito-Avila et al. (2011)
29.	Cotton leafroll dwarf virus	*Gossypium hirsutum*	Illumina Genome Analyzer	sRNAs	Silva et al. (2011)
30.	GVCV	*Vitis vinifera*	Solexa-Illumina platform	sRNA	Zhang et al. (2011)
31.	Rice stripe virus	*Oryza sativa*	Illumina Solexa Sequencer	sRNAs	Yan et al. (2010)
32.	PepMV	*Solanum lycopersicum*	GS FLX Genome Sequencer	cDNA	Adams et al. (2009)
	GMMV				
33.	SPCSV and SPFMV	*Ipomoea batatas*	Illumina Genome Analyzer	sRNA	Kreuze et al. (2009)

Zhang et al. (2011), with the aid of deep and whole-genome sequencing, reported Grapevine vein clearing virus (DNA virus) in six grapevine cultivars linked with the vein-clearing symptom for the first time in Indiana, Missouri, and Illinois, indicating its widespread distribution in the Midwest of the United States. NGS coupled with metagenomic analysis was used to detect Pepino Mosaic Virus and Gayfeather Mild Mottle Virus infecting *Solanum lycopersicum* and *Gomphrena globosa* plant, respectively (Adams et al., 2009).

Ho and Tzanetakis (2014) have developed several barcodes for NGS through the degenerate oligonucleotide-primed RT-PCR method. Moreover, they created a universal bioinformatics tool, VirFind, exclusively for viral detection and discovery. They detected all the viruses in infected samples using this procedure. Such a method also mapped host reads, delivered files of virus reads, and hunted conserved domains for reads of unknown origin. Kehoe et al. (2014) demonstrated that on obtaining complete genomes of viruses through NGS, consideration should be given to sample preparation, efficient genome coverage, and assembly methods. Seguin et al. (2014) reported that deep siRNA sequencing is appropriate for universal identification and classification of evolving viral quasispecies, and to understand fundamental mechanisms behind the biogenesis of siRNA and antiviral defense systems based on RNAi. The near-complete genome sequence of 22 isolates of several different virus species, namely, Potato virus Y, Tobacco vein banding mosaic virus, Cucumber mosaic virus, Tobacco mosaic virus, and *Brassica* yellow virus, were also identified and verified by NGS in infected tobacco plants of Anhui province of China (Akinyemi et al., 2016). Moreover, the discovery and identification of ssRNA viruses is accomplished by utilizing the ribo-depleted RNA in a *de novo* assembly-based method (Visser et al., 2016). They further suggested that a sequencing of one million reads can give adequate genome coverage, particularly for recognition of closterovirus, belonging to the family Closteroviridae and represented by 17 viral species, most of which cause necrosis and yellowing of phloem in plants (Fuchs et al., 2020).

The combinatorial approach based on NGS and automated viral surveillance and diagnosis (VSD) bioinformatics toolkit provided several workflows for distinct pathogenic viruses and viroids that facilitated the surveillance and diagnosis of viral pathogens in plants (Barrero et al., 2017). Multiple reports have proved the utility of NGS for viral detection and identification. Pecman et al. (2017) employed Illumina sequencing to identify and detect plant viruses by comparing RNA sequences of small RNAs with ribo-depleted RNA. The ribo-depleted RNA-based generated datasets were used to identify the putative novel Cytorhabdovirus, due to the reduced number of short reads in the latter. On the contrary, higher yields of viruses and viroid sequences were reported in sRNA pool with no RNA replicative intermediates. Rott et al. (2017) demonstrated the efficiency of NGS by comparing 178 infected tree fruit specimens by conventional as well as NGS methods. NGS was deemed an advanced tool for the identification of novel or poorly characterized viruses relative to traditional bioassays. Bomer et al. (2018) detected the genomes of novel isolates of genera *Badnavirus* and *Potyvirus* by NGS in *Dioscorea* spp. propagated by a robust tissue culture technique. The NGS confirmed its utility in diagnosing yam viruses, contributing towards the safe movement of germplasm between different countries. Liu et al. (2018) sequenced small RNAs by NGS in seven sunflower varieties imported from the United States and the Netherlands. After analysis, a novel endornavirus of double-stranded RNA molecule was detected in two sunflower varieties, X3939 and SH1108. High-throughput sequencing (HTS) goes hand in hand with bioinformatics algorithms for detecting viruses with a higher sensitivity rate. Different algorithms have been employed with HTS to detect twelve plant viruses through small RNA sequencing from three different infected plants (Massart et al., 2019). The virus detection sensitivity ranged from 35 to 100%, reproducibility was 91.6% and the false-positive detection rate was poor. High-throughput sequencing also significantly unveiled the presence of 03 isolates of *Potato virus M* in tomato plants in Slovakia (Glasa et al., 2019). One viroid and eight viruses have also been reported by sequencing of sRNA libraries from infected *Prunus persica* cv. *Nectarina* tree (Xu et al., 2019). In tomato plants, the 10 most abundant sequence variants of potato spindle tuber viroid RG1, differentially expressed with varying time periods, were identified by HTS and thereafter analyzed by employing *in silico* analysis for viroid derived small RNAs (vd-sRNA) (Adkar-Purushothama et al., 2020b). Other studies employing NGS for tomato crops have been reported. Mahmoudieh et al. (2020) surveyed tomato fields from different Peninsular Malaysian regions for the presence and distribution of begomoviruses, Tomato yellow leaf curl Kanchanaburi virus and Pepper yellow leaf curl Indonesia virus by an ORF-based study. They also recognized a novel virus, Ageratum yellow vein Malaysia virus, and its genome of single-stranded DNA and betasatellite component obtained by using NGS showed the maximum sequence similarity with Ageratum yellow vein virus (99%) and Pepper yellow leaf curl betasatellite (91%), respectively. Further, Uehara-Ichiki et al. (2020) detected broad bean wilt virus 2, asparagus virus 2, gaillardia latent virus and tomato spotted wilt orthotospovirus by NGS and RT-PCR analysis in *Valeriana fauriei* Briq. Chiapello et al. (2020) evaluated about 16 libraries from 150 grapevine cultivars infected with *Plasmopara viticola* to characterize the virome associated with the oomycete pathogen. Many plant virus variants including a new ilavirus were detected in grapevine.

NGS discovered four new viruses, namely Camellia yellow ringspot virus (CaYRSV), Camellia chlorotic ringspot viruses (CaCRSVs), Camellia-associated marafivirus (CaMaV), and Camellia-associated badnavirus (CaBaV) in Chinese *Camellia japonica* plants. These studies led to the validation of CaCRSVs as a novel genus belonging to family *Fimoviridae*. On the other hand, CaBaV, CaYRSV, and CaMaV have similar genome organizations and sequence homology with known viruses of the genera *Idaeovirus*, *Badnavirus,* and *Marafivirus*, respectively. In addition, other known viruses such as geminivirus, bluner virus, and betaflexi viruses which existed in the form of heterogeneous mixtures were also detected (Zhang et al., 2020).

Moreover, it is reported that viral pathogens (variants) of green crinkle and apple russet ring are precisely identified by sequencing methods (Li et al., 2020). These studies led to the confirmation that one of the apple chlorotic leaf spot virus sequence variants infects apple to cause distinctive ring-shaped rust, and in addition, the apple stem pitting virus sequence variant was found to cause green crinkle on the fruits of infected apple plants (Li et al., 2020). Based on HTS technology results, Olmedo-Velarde et al. (2020) predicted that viroid-like RNAs (Vd-LRNAs) are correlated with fig trees. Molecular characterization showed that one of the RNAs was a circular RNA with a size ranging from 357 to 360 nucleotides. The biochemical and structural characteristics of this fig hammerhead viroid-like RNA (FHVd-LR) are noticeably identical to those earlier recorded for viroid-like satellite RNAs (Vd-LsatRNAs) and certain viroids. Further studies revealed that FHVd-LR is a unique viroid or Vd-LsatRNA. In accordance with the HTS results, the co-existence of FHVd-LR of dissimilar sizes inside the same host cannot be expelled. Bejerman et al. (2020) reported 70 new plant viral species belonging to negative-sense, single-stranded RNA virosphere by expertly reviewing the application of HTS approaches. It may further be noted that the viral families such as *Aspiviridae*, *Fimoviridae*, *Phenuiviridae*, *Rhabdoviridae*, and *Tospoviridae* include negative-sense and ambisense RNA (NSR) plant viral genomes. NGS-based techniques along with bioinformatic algorithms and (RT)-PCR-based assays have a large impact on viral discoveries by determining the viral genomic sequences, and thus authenticating its reliability in accurately detecting viruses infecting plants. Updated accounts pertaining to the potential of NGS-based high throughput sequencing provide a landmark in the deciphering of detailed information regarding the discovery of viromes to pave way for implementation of genome editing tools, especially CRISPR based tools to develop resistance against harmful viruses. The proceeding section will discuss a detailed and updated account on CRISPR mediated genome editing of desired plant species to develop resistance against economically important plant viruses.

## CRISPR-Based Technologies for Plant Virus Interference

A viral infection can cause up to 98% crop damage in most subtropical and tropical countries, which largely contributes to the global food crisis (Czosnek and Laterrot, 1997). Subsequently, to control the threat of viruses effectively, it is obligatory to boost immunity in crop plants to develop crop resistance to viruses. Over the last decade, limited success has been accomplished through conventional approaches to establish complete resistance against plant viruses. Molecular plant breeding could help in generating resilient plants, which are immune, resistant, or tolerant to viruses. Class II bacterial immune system-based CRISPR/Cas approaches have been extensively implemented and exploited for the modification and detection of nucleic acids (Garcia-Doval and Jinek, 2017; Ji et al., 2019; Cao et al., 2020). The editing of plant genomes based on CRISPR/Cas systems has advanced quickly in the direction of improving plants against devastating viruses. Viral resistance can be achieved in two ways, either by targeting host plant factors that are responsible for replication of the viruses, or by destroying the viral genome and hence inhibiting viral replication (Borrelli et al., 2018; Mushtaq et al., 2020; Table 2).

**TABLE 2:** Successful applications of CRISPR/Cas-mediated genome editing for enhancing plant resistance against viruses.

Targeting viral genomes using CRISPR/Cas-approaches					
Plant species	Target region	Disease	Type of virus	Type of CRISPR variant	References
Arabidopsis, *N. benthamiana*	CP, Rep, IR	BSCV	DNA	Cas9	Ji et al. (2015)
	RBS, 3x Rep, IR hairpin	Bean Yellow Dwarf Virus	DNA	Cas9	Baltes et al. (2015)
	IR, CP, RCRII of Rep	CMV/TMV (TYLCV)	DNA	Cas9	Ali et al. (2015)
	R, CP, RCRII of Re	Geminiviruses (CLCuKoV, MeMV)	DNA	Cas9	Ali et al. (2016)
	1A,CP, 3’UTR-A	CMV/TMV (TYLCV)	RNA	FnCas9 ˃ RNA	Zhang et al. (2018a)
	Hc-Pro, CP	TuMv	RNA	Cas13a	Aman et al. (2018a)
	IR, C1	CLCuMuV	DNA	Cas9	Yin et al. (2019)
Tomato	Rep, CP	TYLCV	DNA	Cas9	Tashkandi et al. (2018)
Potato	CI, Nib, CP, P3	PVY	DNA	Cas13a ˃ RNA	Zhan et al. (2019)
Barley	Rep/RepA, LIR, MP/CP	WDV	DNA	Cas9	Kis et al. (2019)
Rice	RNA	Southern rice black-streaked dwarf virus	RNA	LshCas13a	Zhang et al. (2019)
Targeting a host genome using CRISPR/Cas-approaches					
Arabidopsis	*AtEIF(iso)4E*	TMV	RNA	Cas9	Pyott et al. (2016)
Cucumber	*CseIF4E*	CVYV, ZYMV, PRSV-W	RNA	Cas9	Chandrasekaran et al. (2016)
Rice	*OseIF4G*	RTSV	RNA	Cas9	Macovei et al. (2018)
*N. benthamiana*	*CLC-Nb1a/b*	Reduced PVY intracellular replication	RNA	Cas9	Sun et al. (2018a)
Cassava (CBSV)	*MenCBP-1/2*	CBSV	RNA	Cas9	Gomez et al. (2019)
*Glycine max* (L.) Merr.	*GmF3H1/2, FNSII-1*	SMV	RNA	Cas9	Zhang et al. (2019)
*M. balbisiana*	ORFs of eBSV	Banana streak virus	DNA	Cas9	Tripathi et al. (2019)

Caulimoviridae and Geminiviridae are the most devastating DNA virus families infecting plants comprising single-stranded DNA, as well as those with double-stranded DNA genomes. Numerous independent studies have intended to specifically target and obliterate the genomic DNA of plant caulimoviruses or geminiviruses using genome-editing tools. Before the advent of the CRISPR/Cas systems, the zinc finger nucleases (ZFNs) and transcription activator-like effector nucleases (TALENs) were grossly applied practical technologies to modify the plant host and viral DNAs. TALEN and ZFN mediated gene targeting in geminiviruses, including tobacco curly shoot virus and tomato yellow leaf curl China virus, resulted in viral resistant plants (Chen et al., 2014; Cheng et al., 2015). In contrast to ZFNs and TALENs, CRISPR/Cas systems are a more advantageous, easy, and promising tool for engineering plant resistance to viruses. The key to the susceptibility factor for plant-virus interactions lies in a versatile initiation factor 4E (eIF4E), a component of translation machinery in plants. Plant genomes harbor several recessive viral resistance genes, which encode up to 14 eukaryotic translational initiation factors (eIFs), such as, eIF4E, eIF4G, and other associated proteins. Cloning analysis of all these 14 viral resistant genes revealed that 4E (eIF4E) or its isoform eIF(iso)4E was coded by 12 genes (Wang and Krishnaswamy, 2012). Disruption of the eIF4E gene provides innate immunity to multiple potyviruses in different plant organisms. Following this information, CRISPR/Cas9 has been exercised to produce eIF4E-edited cucumber plants resistant to papaya ringspot mosaic virus-W and zucchini yellow mosaic virus (Chandrasekaran et al., 2016). Likewise, CRISPR/Cas9-based targeting of eIF4G in rice resulted in tungro spherical virus resistant rice plants (Macovei et al., 2018). In another study, Bastet et al. (2019) implemented the conversion of C >G (N176K) to the wild form eIF4E1 in *Arabidosis thaliana* with a cytidine base editor, subsequently resulting in resistance to clover yellow vein virus. Yoon et al. (2020) used CRISPR/Cas9 to produce targeted mutagenesis to determine whether eIF4E1 mutations in *Solanum lycopersicum* cv. Micro-Tom could impart resistance against potyviruses. Genotypic study of the eIF4E1-edited tomato plants in T_0_, T_1_, and T_2_ generations illustrated that these mutations are transmissible to successive generations, and effectively confer resistance to PepMoV. Consequently, these investigations validated the applicability of CRISPR/Cas9 to augment the development of high-quality tomato crops for higher yield and biomass. Atarashi et al. (2020) demonstrated CRISPR/Cas9-mediated mutagenesis in the eIF4E1 gene of a commercial tomato cultivar. In addition to eIF4G, two deletions of three and nine nucleotides (3DEL and 9DEL) and a single nucleotide insertion (1INS) were found in close proximity to regions encoding amino acid residues essential for binding the 5՛ mRNA cap structure. In agreement with earlier studies, inoculation tests with potato virus Y (genus Potyvirus) resulted in substantially reduced susceptibility to the N strain (PVYN), but not to the ordinary strain (PVYO), in 1INS plants. Results propose that genome editing could lead to additional resistance in contrast to mutation breeding. Editing of eIF4E alleles presents an alternative way to control CMV in tomato plants. They performed artificial mutagenesis in the eIF4E1 gene of a commercial tomato cultivar by utilizing CRISPR/Cas9. The recent successful recessive antiviral-type resistance approaches to potyviruses and associated plant viruses are largely based on eIF4s and their homologs. Consequently, there is a need to identify more host susceptibility genes, which can be used as valuable genetic resources to combat economically vital plant viruses. CRISPR-mediated biomimicking mutations in Arabidopsis gene eIF4E1 led to the development of resistance against ClYVV (Bastet et al., 2019). Geminiviruses are known to cause significant losses to commercially valuable crops such as tomato, pepper, and sugarbeet (Langner et al., 2018). Several investigations were conducted for directly targeting the genomic DNA of geminiviruses via CRISPR/Cas9 approaches (Cao et al., 2020; Kalinina et al., 2020). Constructs containing sgRNAs that target the intergenic region (IR) and Rep (replication-associated protein) gene in beet severe curly top virus and bean yellow dwarf virus have been transformed into *Arabidopsis thaliana* and *N. benthamiana* respectively. The subsequent plants displayed a higher degree of resistance against the target viruses (Baltes et al., 2015; Ji et al., 2015). Undoubtedly, CRISPR-based genome editing tools can be effectively engineered to provide specific resistance towards geminiviruses in plants, but Ji et al. (2015) have shown in Arabidopsis plants that the importance of such an antiviral approach is compromised by the off-targeting observed by deep sequencing. They built two virus-inducible CRISPR/Cas9 vectors, which effectively inhibited the aggregation of Beet severely curly top virus in transient assays from transgenic lines of *Nicotiana benthamiana* and Arabidopsis. Deep sequencing did not detect any off-target mutations in the resulting transgenic Arabidopsis lines. Such types of virus-inducible gene-editing methods should be extensively used for designing virus-resistant plants exclusive of off-target costs. Similarly, Tomato and *N. benthamiana* plants expressing gRNA for coat protein or Rep sequences of tomato yellow leaf curl virus exhibit considerable resistance against viruses (Tashkandi et al., 2018). Further studies recorded that the use of CRISPR/Cas9 confers resistance against the wheat dwarf virus in barley plants and banana streak virus in banana plants by targeting conserved coding sequences present in the genome of the viruses (Kis et al., 2019; Tripathi et al., 2019).

**FIGURE 4:**
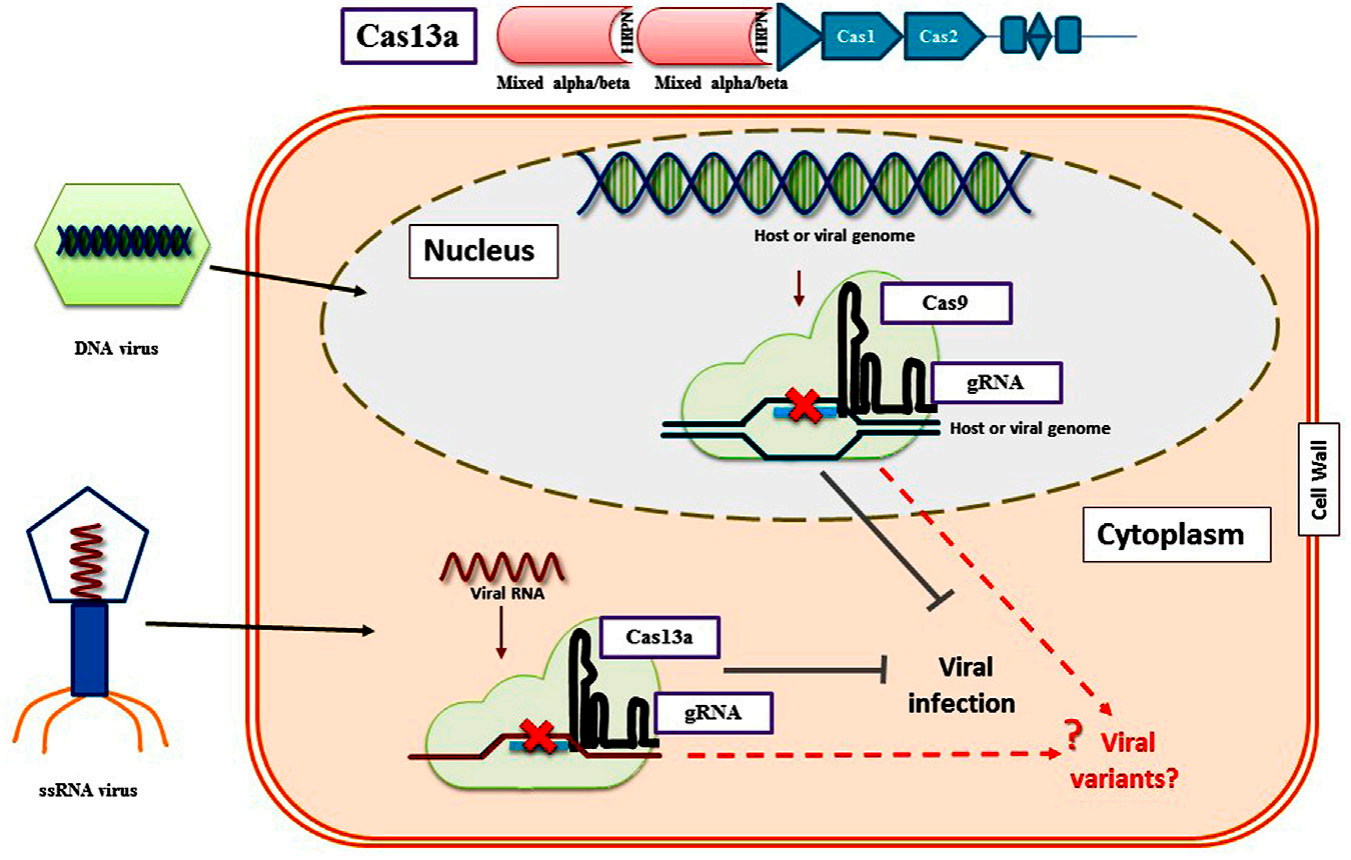
RNA virus interference via CRISPR/Cas13a system in plants. These Cas13 proteins target RNA molecules instead of DNA. Cas13a guided by a crRNA containing a 28-nt spacer sequence cleaves target ssRNAs *in vitro* and *in vivo* with a protospacer flanking sequence (PFS) of A, U, or C, CRISPR/Cas13a knockdown the viral RNA located in plant nuclei. Cas13a can also target multiple RNA transcripts simultaneously using different crRNAs. Moreover, CRISPR/Cas13a system could be used to target a specific RNA in a particular tissue by providing a tissue-specific promoter.

Recently, RNA viruses have been targeted by Cas proteins that include Cas13a from *Leptotrichia shahii* and FnCas9 from *Francisella novicida*. These proteins target RNA molecules instead of DNA. The LshCas13a protein fused with the protospacers could be designed to knock down particular bacterial mRNAs (Abudayyeh et al., 2017). Researchers have engineered CRISPR/Cas13a machinery for *in planta* expression against different plant viruses (Figure 4). Aman et al. (2018a) successfully targeted the tobacco mosaic virus RNA in *N. benthamiana*. A later group of scientists also corroborated the applicability of CRISPR/LshCas13a to engineer *N. benthamiana* to develop resistance against Turnip mosaic virus (TuMV) (Aman et al., 2018a). These reports further paved the way to successfully engineered genomes of rice and *N. benthamiana* to develop resistance against viruses such as Southern rice black-streaked dwarf virus and TMV respectively (Zhang et al., 2019a). Moreover, in a study carried out by Zhang and co-workers, CRISPR/FnCas9 was used to degrade the cucumber mosaic virus and tobacco mosaic virus genome in transgenic lines of *N. benthamiana* and *Arabidosis thaliana* (Zhang et al., 2018b). The same strategies have been effectively used in rice to combat potato virus Y in tobacco and southern rice black-streaked draft virus (Zhang et al., 2019b). In the latest study conducted by Mahas et al. (2019), different Cas13 variants were characterized in order to identify the most specific interference against RNA viruses *in planta* in *N. benthamiana*. They demonstrated that CRISPR-Cas13a system confers modest interference against RNA viruses. High interference activity of LwaCas13a, PspCas13b, and CasRx variants was reported against RNA viruses in transient assays.

In addition, the new Cas13 protein from *Ruminococcus flavefaciens* is classified as a type VI-D effector, called Cas13d (CasRx). Researchers established that Cas13d is advantageous over Cas13a, Cas13b, and Cas13 variants when used to target the CP, GFP, or HC-Pro region in the TuMV-GFP genome (Mahas et al., 2019). In a similar way, Cas13d has also been used in mammalian cells against novel coronavirus SARS-CoV-2 and influenza (Abbott et al., 2020).

Even though CRISPR/Cas-mediated genome editing is applied successfully to control the viral dissemination in transgenic plants, the probable risk of different virus escape events against the CRISPR/Cas9 cleavage results in resistance breakdown caused by the evolution of mutant viruses. Mehta et al. demonstrated that amid 33 and 48% of genome, edited viruses contain a conserved single base-pair mutation that imparted resistance against cleavage by CRISPR/CAS9 system, ensuing resistance breakdown to African cassava mosaic virus (ACMV) during inoculations in greenhouse conditions. While these novel variants of ACMV created by CRISPR/Cas9 mutagenesis might not multiply themselves, they depend on wild-type ACMV to proliferate in *N. benthamiana* (Mehta et al., 2019). The combination of two gRNAs, particularly at distance from each other, would significantly delay resistance breakdown in comparison to using only one sgRNA (Liu et al., 2018).

Thus, risks of virus escape from CRISPR-based antiviral immunity in plants predicts that this mechanism could be viewed as a double-edged sword for providing antiviral engineering. As it can destroy the genome of viruses to inhibit viral infection of crops, it poses a significant problem in that new variants of virus species might be created as by-products of genome editing, suggesting that it will increase the evolutionary process of viruses, or that evolved CRISPR-modified crops may lose their precise resistance to viral pathogens (Lassoued et al., 2019).

One essential aspect for the successful management of a disease is to detect the causal agent rapidly and with accuracy. Plant viruses are known for causing grave economic losses and pose a severe risk to agricultural sustainability. Therefore, optimization of the rapidity, sensitivity, practicability, portability, and precision of virus detection is urgently needed. Recent advances in genome editing technologies have shown that CRISPR-based systems, for example, Cas12a, Cas13a, and Cas14, encompass characteristics which can be used in the detection of nucleic acid (Gootenberg et al., 2017; Chen et al., 2018; Harrington et al., 2018). Cas12a possesses a DNase activity, which can randomly cut nonspecific ssDNA molecules into single/double nucleotides (Chen et al., 2018; Li et al., 2018a; Paul and Montoya, 2020). Researchers from the previous couple of years have used CRISPR-Cas9 protein variants, Cas12a and Cas13 to build easy, convenient, reliable, and economical platforms for nucleic acids detection at the molar level. The Zhang laboratory has exploited ribonuclease activity of the Cas13 protein to establish and refine the technique called Specific High Sensitivity Enzymatic Reporter UnLOCKING (SHERLOCK and SHERLOCKv2) (Gootenberg et al., 2017, Gootenberg et al.,2018). Whereas, Doudna’s laboratory has exploited non-specific ssDNA degradation of Cas12a to establish a process referred to as DNA Endonuclease Targeted CRISPR *Trans* Reporter (DETECTR) (Chen et al., 2018). Both these nucleic acid detection tools exploited the promiscuous cleavage and degradation of adjacent ssRNA and ssDNA using Cas13 and Cas12a, to cleave and activate a reporter. Researchers demonstrated that the SHERLOCK and DETECTR showed the utmost sensitivity and accuracy for the detection of pathogenic viruses (Myhrvold et al., 2018; Chaijarasphong et al., 2019), transgenes (Zhang et al., 2020), and microorganisms (Zhang et al., 2020a).

Co-infection of apple trees with some viruses and viroids is widespread and declines the quality and yield of fruits. Rapid identification of viral pathogens with precision aids in the prevention of virus spread and reduces losses. Existing molecular tests used for the detection of apple viral pathogens involve specialized and costly apparatus. Jiao et al. (2020) optimized a CRISPR/Cas12a based detection approach for the identification of foremost prevailing RNA viruses or viroids in apple, namely apple necrotic mosaic virus, apple stem grooving virus, and stem-pitting virus. Each RNA virus was detected directly from raw leaf extracts following high specificity reverse transcription-recombinase polymerase amplification (RT-RPA). Nevertheless, this procedure was rapid and simple, requiring only about an hour to analyze the leaf samples. This innovative Cas12a-based approach is ideal for rapid and accurate identification of viruses in apple orchards, without sending samples to a specialist laboratory.

**FIGURE 5:**
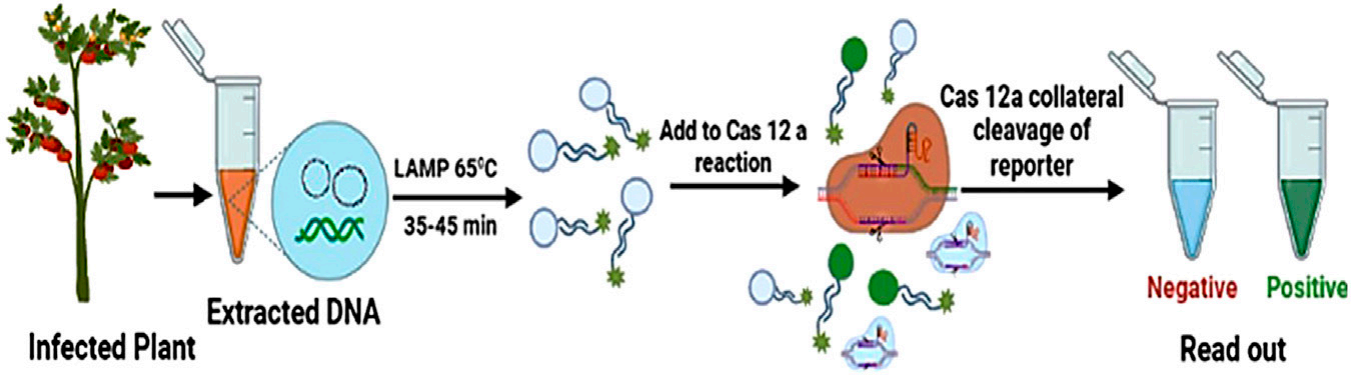
LAMP-coupled Cas12-based assay for the detection of plant viruses. Viral DNA extracted from an infected tomato plant is amplified by loop-mediated isothermal amplification (LAMP), followed by CRISPR-mediated detection. Cas12a-based detection of the LAMP product triggers collateral cleavage of the reporter, thus producing a signal for visual detection.

The above-mentioned CRISPR-based diagnostic tools involve the isothermal amplification of a target sequence, followed by detection of a target using Cas12 in DETECTR or Cas13 in SHERLOCK techniques and the collateral cleavage of a DNA or RNA reporter to specify the presence of the target (Kellner et al., 2019). Regardless of its extensive use for uncovering various pathogens in animals and humans (Li et al., 2019; Van Dongen et al., 2020). Mahas et al. (2020) reported the development and confirmation of a CRISPR-based nucleic acid diagnostic method exploiting the CRISPR–Cas12a system for detecting two geminiviruses, tomato yellow leaf curl virus (TYLCV) and tomato leaf curl New Delhi virus (ToLCNDV). They were successful in detecting TYLCV and ToLCNDV in infected plants with high sensitivity and specificity. This novel nucleic acid detection system can be used to perform an assay in approximately 1 h and makes available easy-to-infer visual readouts by the use of a simple, inexpensive fluorescence visualizer, thus providing an appropriate technique for point-of-use applications (Figure 5). Various reports have established the direct LAMP (loop-mediated isothermal amplification)-based amplification of viral sequences from crude extracts (Panno et al., 2020). This study demonstrates that the LAMP-coupled Cas12a technique is a valid rapid diagnostic tool for plant DNA viruses. Consequently, further upgrading of the LAMP-coupled Cas12a method could make possible the development of this assay as an in-field diagnostic test. The critical advantage of CRISPR-based genome editing lies in its lower off-targeting property to modulate crop genomes for antiviral detection against viruses. The aforementioned reports clearly demonstrate the potential of CRISPR-based genome-editing systems as versatile, efficient, and precise strategies to develop robust antiviral immune systems in crop plants.

## Conclusion and Future Directions

Recent approaches applied in virology are deeply influenced by the technical inputs of NGS technologies. Various sequencing platforms and sample preparation methods used worldwide in research laboratories have led to advancements in the detection and diagnosis of viroids and plant viruses. The last decade witnessed frequent involvement of NGS technologies, even though we also rely on alternative technical practices, preferably for characterization of different viruses. In the past, ELISA (1980s) and later PCR-based techniques (1990s) played the predominant role in detecting viral invasions and diagnosis of disease. However, NGS has facilitated the detection, investigation, and characterization of novel plant viruses that differentiates it from conventional diagnostic tools. The latest forms of NGS technologies, for instance, PacBio by Illumina, Oxford Nanopore, and ISS could be applied to considerably improve plant virology by offering rapid and more reliable viral detection with better precision. The use of HTS for viral diagnostics and the effect of this technique as a significant platform used in the detection of novel viruses have been thoroughly investigated. While different biotechnologies have their benefits and drawbacks, still we are in the infancy of utilizing the full capacity of RNAi and CRISPR/Cas in developing resistance against eukaryotic viruses. Despite the problem of GMO regulations, it can be seen that genome editing would be a powerful method for speeding resistance breeding, taking into account the ever-expanded CRISPR toolkit. Later tool kits can induce mutations to promote the generation of virus-resistant crop ideotypes in cases where resilience in natural variation and wild relatives is restricted.

Thus, the CRISPR/Cas method is widely used tool for selective genome engineering related to other editing approaches and has been developed and implemented in a vast range of plants which act as hosts, and in pathogens, to dissect molecular mechanisms responsible for plant-pathogenic interactions and to improve host resistance to both RNA and DNA viruses. Moreover, several reports suggest that the CRISPR/Cas method has the potential to develop genes with gain-of-function and loss-of-function mutants to decipher plant-virus interactions, and reduce the damage caused by harmful viruses in crops plants.

CRISPR-Cas13 could potentially be employed in disease management of plant viroids over transgenics. For instance, potato spindle tuber viroid (PSTVd) replicates in the nucleus of infected plants, and the mature PSTVd is resistant to RNA interference, hence a CRIPSR-Cas13 system could prove a potential genome editing tool in developing plants resistant to PSTVd. CRIPSR-Cas13 is advantageous over RNAi in terms of specificity, and the cleaved RNA may be further processed by RNAi to design plants with better disease resistance. To sum up, CRIPSR-Cas13 is a novel means to knock down RNA with improved specificity in contrast to RNAi, and it may bestow plants with stronger disease resistance because of the synergistic effect of RNAi.

CRISPR/Cas9 prime editors and base editors can be used to achieve correct genome editing of SNP and SNP typed QTLs effectively in plants, offering manifold resistance for viral pathogens. In a recent study the base-editing-mediated gene evolution (BEMGE) approach has been developed. This innovative crop breeding approach has the ability to artificially evolve every endogenous gene in a plant with a tiled sgRNA library related to the target locus in the genome. Therefore, BEMGE is a potential technique for the transformation of functional genes associated with a defense reaction in plants (Kuang et al., 2020).

In conclusion, CRISPR/Cas technology has the ability to investigate the dynamic spectrum of plant-pathogen interactions. Along with the recent transformation of agriculture and plant disease system, we look forward to CRISPR-based tools contributing to the deciphering of plant-virus interactions in the future and the development of plants with durable and broad-spectrum disease tolerance. NGS and CRISPR-Cas nexus have so far played a crucial role in controlling plant viral diseases. In the coming future, fundamental biological concerns for antiviral engineering could be intercepted using CRISPR technologies and the ongoing GMO-related concerns of plant biosafety regulators may be invalidated.
